# Potential clinical application of surface electromyography as indicator of neuromuscular recovery during weaning tests after organophosphate poisoning

**DOI:** 10.5935/0103-507X.20170035

**Published:** 2017

**Authors:** Maria Bernarda Salazar Sánchez, Alher Mauricio Hernández Valdivieso, Miguel Ángel Mañanas Villanueva, Andrés Felipe Zuluaga Salazar

**Affiliations:** 1 Grupo de Investigación en Bioinstrumentación e Ingeniería Clínica, Facultad de Ingeniería, Universidad de Antioquia - Medellín, Colômbia.; 2 Departamento de Control Automático y Centro de Investigación en Ingeniería Biomédica, Universitat Politècnica de Catalunya - Barcelona, Espanha.; 3 Departamento de Farmacología y Toxicología, Escuela de Medicina, Universidad de Antioquia - Medellín, Colômbia.

**Keywords:** Pesticides, Toxic actions, Ventilator weaning, Electromyography, Respiratory muscles, Case reports

## Abstract

This study aimed to explore the usefulness of measuring respiratory muscle activity in mechanically ventilated patients suffering from acute organophosphate poisoning, with a view towards providing complementary information to determine the best time to suspend ventilatory support. Surface electromyography in respiratory muscles (diaphragm, external intercostal and sternocleidomastoid muscles) was recorded in a young man affected by self-poisoning with an unknown amount of parathion to determine the muscle activity level during several weaning attempts from mechanical ventilation. The energy distribution of each surface electromyography signal frequency, the synchronization between machine and patient and between muscles, acetylcholinesterase enzyme activity, and work of breathing and rapid shallow breathing indices were calculated in each weaning attempt. The work of breathing and rapid shallow breathing indices were not correlated with the failure/success of the weaning attempt. The diaphragm gradually increased its engagement with ventilation, achieving a maximal response that correlated with successful weaning and maximal acetylcholinesterase enzyme activity; in contrast, the activity of accessory respiratory muscles showed an opposite trend.

## Abstract (pt)

Este estudo teve como objetivo explorar a utilidade da avaliação da atividade muscular respiratória em pacientes em uso de ventilação mecânica após envenenamento agudo por organofosforados, para fornecer informações complementares para determinação do melhor momento para suspensão do suporte ventilatório. Foi registrada eletromiografia de superfície em músculos respiratórios (diafragma, intercostais externos e esternocleidomastóideos) em um homem jovem afetado por autoenvenenamento com quantidade desconhecida de paration, para determinar o nível de atividade muscular no decurso de diversas tentativas de desmame da ventilação mecânica. A distribuição de energia de cada frequência de sinal de eletromiografia de superfície; a sincronização entre máquina, paciente e músculos; a atividade da enzima acetilcolinesterase; o trabalho respiratório e os índices de respiração rápida e superficial foram calculados em cada uma das tentativas de desmame. O trabalho respiratório e o índice de respiração rápida e superficial não se correlacionaram com a falha ou o sucesso da tentativa de desmame. O diafragma aumentou gradualmente seu envolvimento com a ventilação, tendo alcançado resposta máxima, que se correlacionou com o sucesso do desmame e a atividade máxima da enzima acetilcolinesterase. Por outro lado, a atividade de músculos respiratórios acessórios mostrou tendência oposta.

## INTRODUCTION

Organophosphorus compounds (OC) are esters of phosphoric acid that are mainly used as agricultural pesticides or as chemical weapons. Acute organophosphate poisoning remains a major health problem related to careless manipulation, self-poisoning and chemical warfare. In Colombia, three large-scale pesticide poisonings have occurred in the last 50 years and have involved at least 1000 persons, with a mortality of nearly 10%.^(1)^ Worldwide, fatal respiratory failure secondary to this type of poisoning requires the use of mechanical ventilators as a supportive measure, requiring approximately 1.7 million days of ventilation every year.^(2)^ Weakness, paralysis and failure of ventilation can be related to depression of central respiratory centers but are more commonly associated with the overstimulation of nicotinic receptors. The treatment of OC-poisoned patients involves the continuous evaluation of the respiratory function. However, to our knowledge, there is no systematic and controlled procedure to identify the proper time to start spontaneous breathing tests (weaning procedures) after an OC poisoning.

The present article describes the use of surface electromyography to monitor the respiratory muscle activity after neuromuscular blockade in real time in a young man who was affected by self-poisoning with an unknown amount of parathion and to define its correlation with the success of the weaning process.

## CASE REPORT

A twenty-eight-year-old man, weighing 70kg and 175cm in height, was brought to the Emergency Department at the *Hospital Universitario San Vicente Fundación* in Medellin, Colombia. He arrived one and a half hours after drinking an unknown amount of organophosphorus pesticide during a suicide attempt. On admission, gastric lavage and activated charcoal were given promptly. Initially, he had miosis, bronchorrhea, frequent urination, a heart rate of 110 beats per minute and a blood pressure of 109/65mmHg. To counteract the cholinergic syndrome, a bolus of 1mg of atropine was administered i.v., followed by additional boluses every 5 minutes until the patient's condition was stabilized. A total amount of 37mg of atropine was used to achieve a reduction of secretions and respiratory distress. An electrocardiogram showed sinus tachycardia with prolongation of the corrected QT interval (520ms). A few hours later, the patient had a dramatic decrease in carbon dioxide removal, attaining a partial arterial pressure of carbon dioxide of 57mmHg and a blood pH of 7.08, which suggested acute respiratory acidosis. At this moment, the acetylcholinesterase enzyme (AChE) activity in red blood cells was 2.44% (0.0219 ∆pH/hour). The prolonged altered mental status plus an oxygen partial pressure (PaO_2_) < 60mmHg prompted the admission of the patient into the intensive care unit (ICU), where he was intubated. Midazolam and fentanyl were intravenously administered during the first 24 hours as sedatives. On arrival to the ICU, the Acute Physiology and Chronic Health Evaluation II (APACHE) score was 34. The time to start the spontaneous breathing test was defined by clinical expertise plus the results of measurements of several ventilatory signals used as classical predictors (i.e., work of breathing - WOB), but the lack of any of the following previous conditions was assessed: hypoxemia, mental status, cardiac arrhythmia and respiratory distress. The patient was ventilated in spontaneous mode during each weaning test, with a positive end-expiratory pressure (PEEP) level of 5cmH_2_O, pressure support of 7cmH_2_O and fraction of inspired oxygen (FiO_2_) of 40%.

The first weaning was attempted on the second day. The patient initially tolerated the test but showed miosis and bronchorrhea (poisoning symptoms) shortly thereafter; therefore, the patient could not be extubated. The plasma AChE activity was 447U/L (9.60%). On the eleventh day, the patient did not tolerate a second spontaneous breathing test, showing signs of agitation and respiratory distress. The AChE activity remained reduced, with a value of 1,646U/L (35.3%). On the thirteenth day, a third unsuccessful attempt at extubation was performed, but 48 hours later, the patient showed a PaO_2_ < 60mmHg, a carbon dioxide partial pressure (PaCO_2_) of 72mmHg and a PaO_2_/FiO_2_ ratio of 104, which required re-intubation. A plasma AChE of 3,818U/L (79.8%) was observed. Finally, a successful fourth weaning test was performed on the seventeenth day; the plasma AChE activity was 4,370U/L (93.8%), and the PaO_2_ and PaCO_2_ reached permissible values, 80mmHg and 43mmHg, respectively. After extubation, the patient was discharged from the ICU.

Table 1 shows the following ventilatory function parameters used as classical predictors of weaning. The median of the WOB minimally decreased from 0.39J/L to 0.26J/L between the first and last weaning attempts, respectively. Regarding rapid shallow breathing (RSB), all of the values incorrectly suggested that all weaning tests were likely to be successful (RSB < 100).

**Table 1:** Measurements during spontaneous weaning tests

Weaning test attempt	Days in ICU	Outcome	AChE (%)	WOB (J/L)	RSB (breaths/minute)
First	2	Failed	9.60	0.39 ± 0.04	29.5 ± 3.22
Second	11	Failed	35.3	0.43 ± 0.11	62.7 ± 17.1
Third	13	Failed	79.8	0.47 ± 0.21	52.5 ± 9.29
Fourth	17	Successful	93.8	0.26 ± 0.09	48.7 ± 7.94

ICU - intensive care unit; AChE - the plasma acetylcholinesterase activity; WOB - work of breathing; RSB - rapid shallow breathing.

### Respiratory muscle activity

Table 2 summarizes the squared Pearson correlation coefficients (R^2^) between muscles and between patient and machine, and the ratio between energy in high and low frequencies (RHL). The R^2^ indicates the grade of linear correlation between muscles or between patient and machine in each weaning test. Values of RHL higher than 250 were observed for the external intercostal (Extint) and in the three muscles during the first and second weaning attempts, respectively, which could be attributed to an increase in the activity of respiratory muscles. Regarding the synchronization between pairs of muscles and the machine-patient, different muscle coordination levels were observed. In fact, a variable and low correlation coefficient was determined between the first and third weaning tests, which suggested poor synchronization in the first weaning test between the diaphragm (Dia) - sternocleidomastoid (Strn) and diaphragm-external intercostal. In contrast, the paired airway pressures (P_aw_)-Extint and P_aw_-Strn showed better synchronization. This result suggests that accessory respiratory muscles (external intercostal and sternocleidomastoid) were more engaged than the diaphragm was. During the second and third attempts, there was an increase in muscle coordination between the diaphragm and the other two muscles, despite the low correlation coefficient between muscles and airway pressure showing lower machine-patient engagement.

**Table 2:** Mean values for relevant variables during respiratory cycles of the studied weaning tests

Variables		Weaning test attempt			
		First	Second	Third	Fourth
RHL	Diaphragm	150	499	146	225
RHL	External intercostal	774	568	250	98.1
RHL	Sternocleidomastoid	204	1140	67.3	102
Muscle synchronization					
R^2^	Diaphragm, external intercostal	0.04	0.12	0.67	0.20
R^2^	Diaphragm, sternocleidomastoid	0.04	0.21	0.70	0.57
R^2^	External intercostal, sternocleidomastoid	0.82	0.30	0.63	0.16
Synchronization machine-patient					
R^2^	Paw, diaphragm	0.02	0.12	0.20	0.74
R^2^	Paw, external intercostal	0.42	0.18	0.17	0.48
R^2^	Paw, sternocleidomastoid	0.57	0.20	0.24	0.22

The ratio of energy in high and low frequencies evaluated in the diaphragm, external intercostal and sternocleidomastoid muscles. The squared Pearson correlation coefficient between the rectified amplitudes of surface electromyography of two different muscles and the squared Pearson correlation coefficient between the airway pressure and the rectified amplitudes of each muscle. RHL - ratio between energy in high and low frequencies; R^2^ - squared Pearson correlation coefficient; Paw - airway pressure.

**Figure 1:**
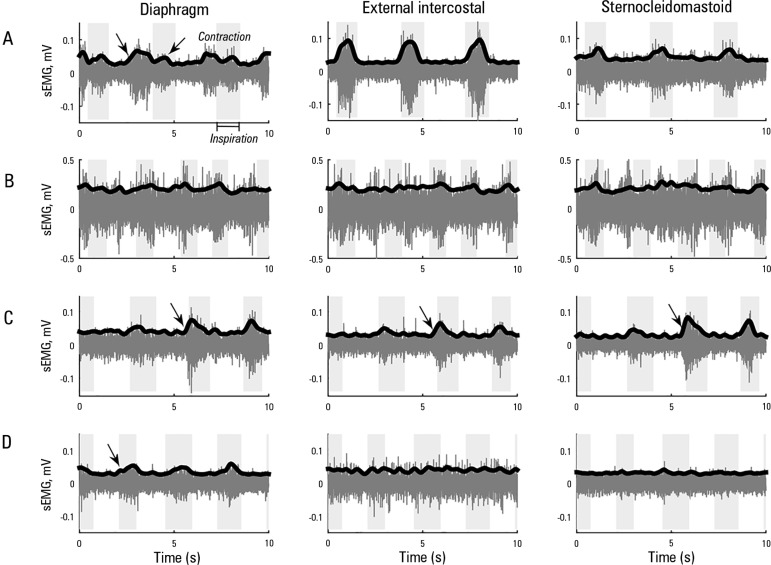
Surface electromyography from the diaphragm, external intercostal and sternocleidomastoid muscles recorded during the weaning tests: (A) first, (B) second, (C) third and (D) fourth attempt. The shaded background area corresponds to the inspiratory phase, and the arrows highlight the muscle contraction. sEMG - surface electromyography.

Figure 1 illustrates the surface electromyography (sEMG) signal of the patient's muscles in the inspiratory and expiratory phase during the spontaneous breathing test. During the second weaning attempt (Figure 1B), there was no contraction in any recorded muscle; in contrast, the muscle activity increased in the third attempt (Figure 1C). Ultimately, the successful weaning was mainly characterized by improved correlations between the diaphragm-sternocleidomastoid and the paired P_aw_-Dia (Table 2). In the fourth attempt, the highest correlation coefficient was for P_aw_-Dia, which was associated with a proper response of the machine to the patient's muscle effort. Thus, as shown in figure 1D, the diaphragm muscle appears to play a more significant role in mechanical ventilation in comparison to the external intercostal and sternocleidomastoid muscles.

## LITERATURE REVIEW

The mechanism of action of these substances involves the inhibition of AChE by a stable OC-AChE bond that leads to the overstimulation of nicotinic and muscarinic receptors.^(3)^ Peripheral markers of organophosphate poisoning, such as red blood cell cholinesterase levels, have been used to determine the response to therapy, but the use and interpretation of these assays remain controversial.^(3)^ The symptoms and signs due to acute stimulation of muscarinic receptors include bronchorrhea, bronchospasms, hypotension, bradycardia, salivation, incontinence, miosis, agitation, confusion, excessive sweating and cramps. Weakness, paralysis and failure of ventilation can be related to depression of the central respiratory center but are more commonly associated with the overstimulation of nicotinic receptors.

Intubation and mechanical ventilation are recommended in patients with any of the following signs: (i) tidal volume lower than 5mL/kg, (ii) vital capacity lower than 15mL/kg, (iii) PaO_2_ less than 60mmHg or (iv) FiO_2_ greater than 60%.^(4)^ To our knowledge, however, there is no systematic, controlled procedure for identifying the proper time to initiate spontaneous breathing tests (weaning procedures) after an OC poisoning. Currently, the weaning procedure is completely dependent on clinical expertise, and it is mandatory to obtain a successful weaning for the full recovery of the patient, because a high risk of failure is associated with imbalances between the ability of the respiratory muscles and the demands of the respiratory control system.^(5)^ Because spontaneous breathing tests do not include quantitative information about the real-time activity of the respiratory muscles, this limitation may contribute to the high failure rate (10 to 20%).^(6)^ Surface electromyography is a non-invasive method that allows information about muscular activity to be collected in real time. There are reports using electromyography available on mechanically ventilated patients^(7)^ but not in patients after poisoning with OC.

The treatment protocol defined in this Hospital for patients under these clinical conditions indicates that weaning tests are only performed if i) the patient passes a security test with suspended sedation, ii) the patients passes a wake-up test during the next four hours after the suspension of sedation and iii) there is no presence of delirium, as evaluated by the Richmond Agitation-Sedation Scale (RASS). Therefore, the weaning test is not necessarily performed daily.

## DISCUSSION

Moon et al. suggested that a significantly lower value for the AChE activity in erythrocytes is associated with the most severe signs in OC-poisoned patients.^(3)^ The AChE activity of the patient upon admission was 2.4%; therefore, the patient's respiratory failure could be attributed to overstimulation of nicotinic receptors.^(2)^ Grubić et al. found in rats that recovery of AChE activity in the diaphragm and brain after the irreversible inhibition was only 50% of the normal value one week after poisoning.^(8)^ The patient exceeded 50% of the normal value thirteen days after intoxication; this difference in time may indicate that recovery depends on the ingested dose of toxin, which was unknown in this case.

Classical indices such as WOB and RSB were not suitable for determining the proper time to withdraw ventilatory support. The WOB values for all weaning tests were close to the average value of a healthy subject, 0.5J/L.^(9)^ Therefore, defining reference values for WOB of patients under this condition may require a deeper understanding of the intricacies of mechanical ventilation, including the effects of pressure support and the level of PEEP on respiratory load. Concerning the rapid shallow breathing, values < 100 breaths/min suggested a high probability of successful extubation for all weaning tests;^(10)^ therefore, establishing whether the intoxicated patient has a pattern of RSB is not sufficient to determine whether the ventilatory support should remain. Karthika et al. found that an RSB lower than 105 was unable to predict the failure of weaning in patients with a different diagnosis who were also under mechanical ventilation.^(10)^ Rapid shallow breathing could be useful if other signs of discomfort, such as sweating and fasciculation, are considered.

Indices obtained from sEMG signals presented here showed that a gradual increase in the response of the diaphragm correlated with weaning test outcome. The patient had activity in the sEMG pattern of the diaphragm during the first weaning, showing contractions during exhalation and the inspiration phase (Figure 1). This activity shows asynchrony between the mechanical ventilator and the patient related to the hyperactivation of nicotinic receptors, which particularly affects the diaphragm.^(8)^ The low correlation coefficients between the air pressure signal and muscle activity (P_aw_-Dia, P_aw_-Extint and P_aw_-Strn) during the second weaning test suggest muscle weakness, which is confirmed by the energy of sEMG signals that shifted to high frequencies.^(11)^ Despite sEMG activity in all muscles, the breaths during this test were mainly supported by the mechanical ventilator because the diaphragm, external intercostal and sternocleidomastoid muscles showed an inability to maintain independent ventilation and an asynchronous breathing pattern. The diaphragm's inability might be attributed to the persistence of muscular blockage, which is consistent with histological studies that found an association between the combination of at least 18 hours of mechanical ventilation and diaphragmatic inactivity with atrophy in the human diaphragm, but not in the pectoral muscles.^(12)^ In contrast to the second test, the coordination and activity of respiratory muscles (Dia-Extint, Dia-Strn and Extint-Strn) increased with an energy shift to low frequencies in the third test,^(11)^ which suggests recovery of the muscles. Concerningly, for the machine-patient synchronization, the R^2^ was as low as the values in the first and second tests, which suggests that despite the muscle recovery found, the engagement of the diaphragm, external intercostal and sternocleidomastoid muscles was not sufficient to maintain spontaneous breathing. This is in agreement with the outcome of weaning tests that required reintubation within the first 48 hours after extubation. In line with later results, authors such as Parthasarathy et al. have found that accessory muscles are recruited proportionally to the diaphragm in intubated patients during failed weaning trials.^(13)^ On the fourth attempt, both the highest correlation coefficient between the diaphragm and the airway pressure (Table 2) and the energy of sEMG signals consolidated at low frequencies suggested a muscular activity close to that observed under normal conditions;^(11)^ i.e., the diaphragm recovered as the main breathing muscle without the need to recruit accessory respiratory muscles.

This case report introduces the use of new sEMG-based variables to overcome the limitations of using only mechanical respiratory variables, according to the activation of not only the diaphragm but other muscles. Interesting findings in this case study were the observation that a progressive increase in the correlation coefficient between the airway pressure and diaphragm was related to proper engagement of the main respiratory muscle with spontaneous ventilation from the first to fourth weaning test. The opposite trend occurs with the accessory muscles, which decrease their participation in spontaneous breathing.

## CONCLUSION

Conventional mechanical ventilator indices fail to provide robust and clear information to appropriately handle patients suffering from acute organophosphate poisoning. As a result, extubation procedures are generally conducted in a time frame when the patient is still in need of exogenous ventilatory support. The clinical report presented here introduces an alternative approach to estimate proper extubation times for such patients. We propose to collect muscle activation data via surface electromyography and simple signal analysis to determine the appropriate timing for initiating extubation maneuvers.

The main indices to be recovered from the data include the ratio between energy in high and low frequencies and the correlation coefficients between the muscle activity and the airway pressure that give us an idea of the muscle state, in contrast to the traditional indices that are mainly sensitive to changes in the state of respiratory mechanics. The final goal of collecting and processing this new set of indices is to provide physicians with robust protocols to handle intensive care unit patients with complex muscular blockages.

## References

[1] Idrovo AJ (1999). Intoxicaciones masivas con plaguicidas en
Colombia. Biomédica.

[2] Nelson LS, Lewin NA, Howland MA, Hoffman RS, Goldfrank LR, Flomenbaum NE (2011). Goldfrank's toxicologic emergencies.

[3] Moon J, Chun B, Lee S (2015). Variable response of cholinesterase activities following human
exposure to different types of organophosphates. Hum Exp Toxicol.

[4] Barbas CS, Ísola AM, Farias AM, Cavalcanti AB, Gama AM, Duarte AC (2014). Brazilian recommendations of mechanical ventilation 2013. Parte
I. Rev Bras Ter Intensiva.

[5] Doorduin J, van der Hoeven JG, Heunks LM (2016). The differential diagnosis for failure to wean from mechanical
ventilation. Curr Opin Anaesthesiol.

[6] Thille AW, Cortés-Puch I, Esteban A (2013). Weaning from the ventilator and extubation in ICU. Curr Opin Crit Care.

[7] Dres M, Schmidt M, Ferre A, Mayaux J, Similowski T, Demoule A (2012). Diaphragm electromyographic activity as a predictor of weaning
failure. Intensive Care Med.

[8] Grubić Z, Sketelj J, Klinar B, Brzin M (1981). Recovery of acetylcholinesterase in the diaphragm, brain, and
plasma of the rat after irreversible inhibition by soman: a study of
cytochemical localization and molecular forms of the enzyme in the motor end
plate. J Neurochem.

[9] Cabello B, Mancebo J, Pinsky MR, Brochard L, Mancebo J (2012). Work of breathing. Applied Physiology in Intensive Care Medicine 1:Physiological Notes -
Technical Notes - Seminal Studies in Intensive Care.

[10] Karthika M, Al Enezi FA, Pillai LV, Arabi YM (2016). Rapid shallow breathing index. Ann Thorac Med.

[11] Mañanas MA, Jané R, Fiz JA, Morera J, Caminal P (2000). Study of myographic signals from sternomastoid muscle in patients
with chronic obstructive pulmonary disease. IEEE Trans Biomed Eng.

[12] Levine S, Nguyen T, Taylor N, Friscia ME, Budak MT, Rothenberg P (2006). Rapid disuse atrophy of diaphragm fibers in mechanically
ventilated humans. N Engl J Med.

[13] Parthasarathy S, Jubran A, Laghi F, Tobin MJ (2007). Sternomastoid, rib cage, and expiratory muscle activity during
weaning failure. J Appl Physiol.

